# Premenstrual dysphoric disorder as a potential predisposing factor for Alzheimer’s disease: a review

**DOI:** 10.3389/fendo.2025.1580947

**Published:** 2025-10-14

**Authors:** Jie Yang, Ming Cheng, Zhaoshu Jiang, Chunyu Du, JinNan Zhao, Zhenliang Luo, Zhen Zhang

**Affiliations:** ^1^ Yangsheng College of Traditional Chinese Medicine, Guizhou University of Traditional Chinese Medicine, Guiyang, Guizhou, China; ^2^ Qinhuangdao Shanhaiguan Pharmaceutical Co., Ltd, Qinhuangdao, Hebei, China; ^3^ Department of Integrated Traditional Chinese & Western Medicine, The Second Xiangya Hospital, Central South University, Changsha, Hunan, China

**Keywords:** premenstrual dysphoric disorder, Alzheimer’s disease, neurotransmitters, hormonal fluctuations, neuroinflammation

## Abstract

Premenstrual dysphoric disorder (PMDD) and Alzheimer’s disease (AD) differ significantly in terms of onset period and clinical manifestations. However, recent studies suggest that the two conditions may share potential links at the neuroendocrine and molecular levels. This review synthesizes current research progress and explores the intersecting biological pathways between PMDD and AD, with a particular focus on dynamic fluctuations in estradiol (E2) and allopregnanolone (ALLO), dysregulation of the γ-aminobutyric acid (GABA)ergic system and serotonergic (5-HT) neurotransmitter systems, and sex-specific vulnerability associated with the apolipoprotein E epsilon 4 (APOE ϵ4) allele. These mechanisms suggest that PMDD may serve as a potential biological precursor state for AD, offering valuable implications for early screening and intervention. The analysis provides new theoretical insights and research directions for identifying high-risk female populations, understanding sex differences in AD pathogenesis, and developing targeted therapeutic strategies.

## 1 Introduction

PMDD and AD are representative disorders of emotional (1) and neurodegenerative conditions occurring at distinct stages of life (2). Although the two conditions differ markedly in terms of clinical presentation, age of onset, and disease trajectory, emerging research in neuropsychiatry has begun to uncover potential overlaps in their underlying biological mechanisms, particularly in relation to neurotransmitter system dysfunction, hormonal fluctuations, and chronic inflammatory responses.

However, existing studies have primarily focused on PMDD as a precursor to major depressive disorder (MDD) or anxiety disorders, with limited literature systematically exploring its potential role as an early risk state for AD. In particular, although E2, ALLO, GABAergic and 5-HT systems, and the APOE gene have all been implicated in the regulation of cognition, emotion, and neuroinflammation in both PMDD and AD, integrative analyses of their shared mechanistic roles across the two disorders remain scarce (1, 3, 4). Current literature has yet to adequately identify or systematically elucidate whether PMDD may reflect a sex-specific neurobiological vulnerability that predisposes individuals to AD pathogenesis.

**Figure 1:**
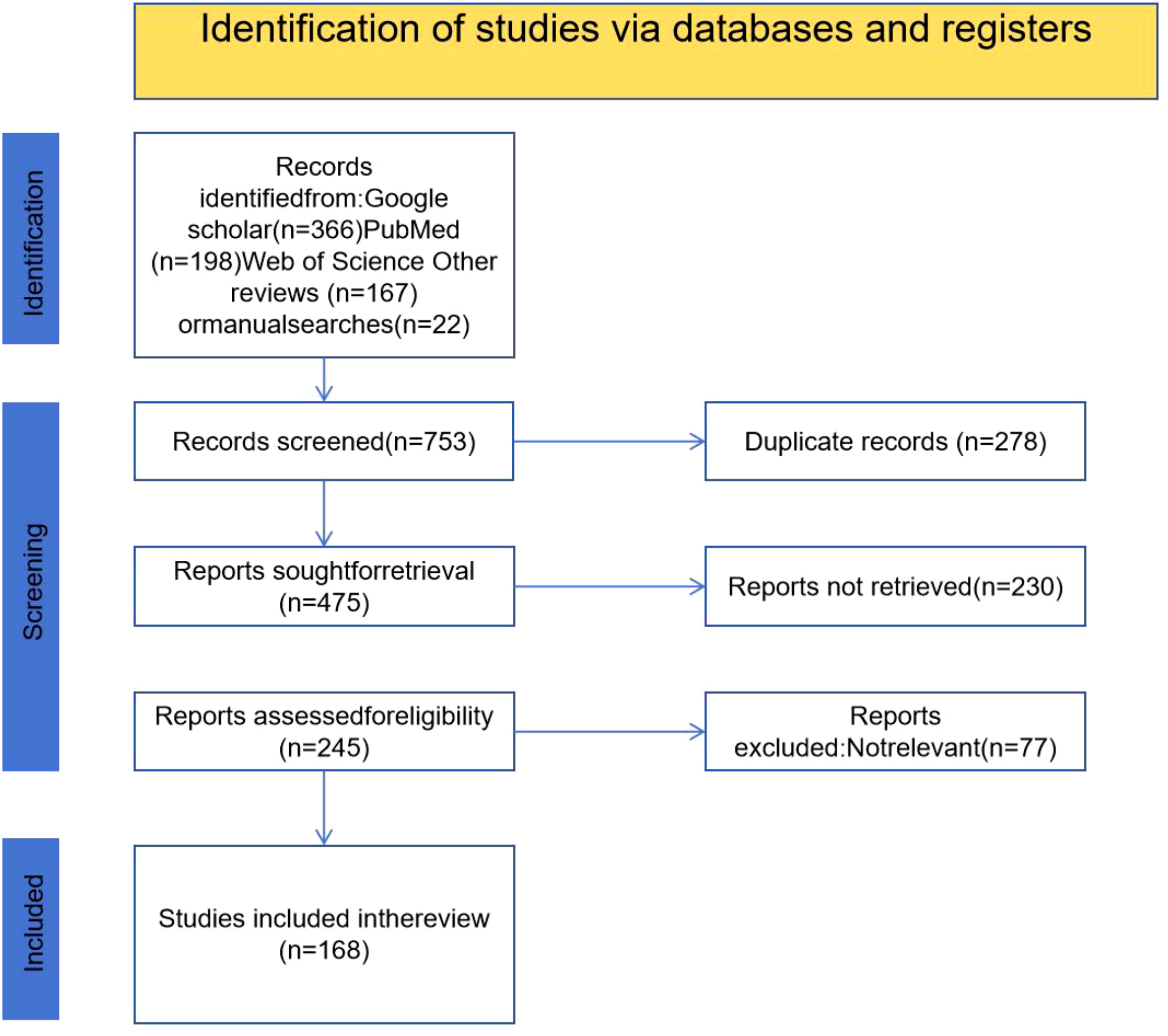
PRISMA flow diagram illustrating the study selection process. A total of 753 records were initially identified through database searches (Google Scholar, PubMed, Web of Science, and other sources). After removing 278 duplicates, 475 records were screened. Of these, 230 reports were not retrieved and 77 were excluded due to irrelevance, resulting in 168 studies being included in the final review.

Therefore, this review aims to build upon current neuroendocrinological and psychopathological research by integrating the roles and interrelationships of five key mechanisms— E2, ALLO, GABAergic system, 5-HT transmission, and the APOE genotype—in the context of both PMDD and AD, thereby attempting to construct a theoretical framework linking the two disorders. By elucidating the potential shared mechanisms between PMDD and AD, this study not only addresses a critical gap in the literature, but also offers new theoretical support for early screening of AD, identification of high-risk populations, and the development of personalized intervention strategies (**Figure 1**).

## 2 Premenstrual dysphoric disorder

### 2.1 Diagnosis and epidemiology of premenstrual dysphoric disorder

PMDD is an emotional disorder listed in the Diagnostic and Statistical Manual of Mental Disorders, Fifth Edition (DSM-5). The diagnostic criteria for PMDD in DSM-5 include 11 symptoms, of which 4 emotional symptoms are core (depressed mood, irritability, anxiety and mood instability), and the remaining 7 are related to cognitive, behavioral, and somatic symptoms (difficulty concentrating, feeling overwhelmed, reduced interest in daily activities, low energy, changes in appetite, hypersomnia or insomnia, and physical symptoms) (5, 6). A survey study by Richards and Oinonen developed a retrospective premenstrual screening questionnaire consistent with DSM-5 criteria for PMDD. The study found that 34% of women in their sample might have PMDD (7). Notably, PMDD diagnoses based on retrospective self-report are fundamentally unreliable and prone to false positives (8), studies have indicated that using retrospective symptom recall for diagnosis may result in a false-positive rate as high as 60% (9). The DSM-5 Text Revision (DSM-5-TR) (10) the first revision since the original DSM-5 was published in 2013, has made revisions to the PMDD criteria. According to DSM-5-TR, the DSM-5-TR (11) provides supplementary clarification and refinement of the diagnostic criteria for PMDD. While the core diagnostic items (A–G) remain unchanged, the revised text significantly enhances clinical applicability and diagnostic precision in several aspects. Major updates include: further emphasizing that symptoms must cause clinically significant social or occupational impairment; mandating at least two cycles of prospective daily symptom ratings, with standardized tools such as the Daily Record of Severity of Problems (DRSP) being recommended; revising the prevalence rate to 1.3% based on more rigorous prospective research; and incorporating new content regarding suicide risk, cultural influences, and responses to hormonal treatments. These changes reflect stricter clinical recognition criteria for PMDD and establish a more standardized diagnostic foundation for future research on underlying mechanisms and population-level screening. Future studies should incorporate large-scale longitudinal cohorts and multi-center samples, utilizing prospective designs and biomarker validation to systematically track symptom fluctuations and relevant neurobiological indicators in PMDD patients, thereby enhancing the objectivity and credibility of PMDD diagnoses.

### 2.2 Pathogenesis of premenstrual dysphoric disorder

The most prominent feature of Premenstrual Syndrome (PMS)/PMDD is the temporal relationship between symptom onset and the menstrual cycle, indicating the influence of gonadal steroids and their metabolites, which play a role in adjusting various biological systems required for reproductive goals (1). PMDD symptoms follow a predictable pattern in relation to menstruation (with symptoms being more severe in the week before menstruation compared to the week after), leading many to hypothesize that estrogen, progesterone, or their combination may trigger emotional symptoms (12). Research on rodents has shown that E2 has proconvulsant effects, accelerating seizures induced by the amygdala (13, 14). A study by Schmidt et al. suggests that abrupt changes in E2 and progesterone levels, from low to high, may be the mechanism behind PMDD symptoms, rather than the steady-state levels causing PMDD onset. This finding is consistent with multiple studies both domestically and internationally and is widely accepted in the academic community (15–17). The 5-hydroxytryptamine (5-HT)ergic system is a plausible neurobiological cause of PMDD.5-HT is a neurotransmitter that plays a key role in mood regulation, and its reduced levels are associated with depressive symptoms (18). In women with PMS/PMDD, serotonergic dysregulation is characterized by atypical neurotransmission, reduced transporter and receptor density, decreased peripheral 5-HT levels during the luteal phase, and elevated levels during the follicular phase (19). Positron emission tomography (PET) can be utilized to observe *in vivo* neurochemical changes and metabolic activity during depressive episodes (20). A PET study (21) demonstrated the direct role of 5-HT in PMDD. The study found a significant correlation between daily mood ratings in PMDD women and changes in ^11^C-labeled 5-HT brain uptake in different brain regions across the menstrual cycle. A study by Brzezinski and Menkes et al. found that the 5-HT agonist fenfluramine improved PMS symptoms, whereas acute depletion of the 5-HT precursor tryptophan exacerbated them (22, 23). Selective serotonin reuptake inhibitors (SSRIs) block the reuptake of 5-HT in the presynaptic cleft, thereby increasing 5-HT concentrations in the synaptic space and enhancing serotonergic neurotransmission. SSRIs are considered first-line therapy for PMDD (24). The rapid effects of SSRIs on PMS/PMDD women may be due to their simultaneous influence on 5-HT receptors and ALLO levels in the brain, thereby indirectly modulating the function of gamma-aminobutyric acid A receptors (GABA_AR) (25, 26). ALLO is a metabolite of progesterone, synthesized in the brain (such as the hippocampus), adrenal glands, and gonads (27, 28),and its presence has been confirmed through human brain tissue and animal studies and its presence has been confirmed through human brain tissue and animal studies (29). In normal reproductive-age women, serum ALLO levels range from 0.2 to 0.5 nmol/L during the follicular phase, increase to 4 nmol/L during the mid-luteal phase (30), and fluctuate between 0.9 and 2 nmol/L during the late luteal phase (31–33). However, compared to the normal luteal phase ALLO levels, both high and low ALLO concentrations are associated with more severe mood changes, suggesting a bimodal or inverted “U” effect of ALLO on mood fluctuations (34). In the nervous system, ALLO is a potent positive allosteric modulator. It binds to specific sites on GABA_AR, enhancing the receptor’s sensitivity to GABA, increasing chloride ion influx, and leading to neuronal hyperpolarization, thus exerting inhibitory effects (35, 36), This mechanism gives ALLO significant sedative, anxiolytic, antidepressant, anticonvulsant, and neuroprotective properties (37). The pathogenesis of PMDD is complex and multifaceted, and is still evolving, thus requiring further in-depth exploration.

## 3 Alzheimer’s disease

### 3.1 Diagnosis and pathogenesis of Alzheimer’s disease

AD is the most common neurodegenerative disease globally, leading to severe cognitive decline and irreversible memory loss (38). The World Health Organization (WHO) predicts that by 2050, approximately 132 million people worldwide will be affected by dementia (39, 40). In 2018, based on the diagnostic framework of the National Institute on Aging and the Alzheimer’s Association, abnormal biomarkers of amyloid β-protein (Aβ) and tau protein (Tau) were defined as AD, even in the absence of cognitive symptoms (41). The 2024 updated guidelines on “Alzheimer’s Disease Diagnosis and Staging” categorize biomarkers into three broad categories: core biomarkers for Alzheimer’s Disease Neuropathological Changes (ADNPC) (42); nonspecific biomarkers that are important in the AD pathogenesis but also involved in other brain diseases; and biomarkers for common comorbidities not related to AD. This update includes three new categories of biomarkers: inflammation/immune mechanisms (I), vascular brain injury (V), and α-synucleinopathy (S) (43). The most prominent morphological feature of AD is the extracellular deposition of Aβ, forming characteristic amyloid plaques. Additionally, hyperphosphorylated tau protein accumulates within neurons, forming neurofibrillary tangles (NFTs), which disrupt the function of the neuronal cytoskeleton (44). APOE is a lipid-binding protein abundant in human plasma, and polymorphisms at the APOE gene locus have been identified as a risk factor for AD (45). The exact molecular mechanisms of AD remain unclear, and there are no effective drugs available to halt or reverse the progression of the disease (46).

### 3.2 The incidence of Alzheimer’s disease is higher in women than in men

There are significant sex differences in the incidence, prevalence, and clinical experience of Alzheimer’s disease (AD), with two-thirds of patients being women (47), Multiple biological mechanisms may be involved, including hormones, pregnancy, brain structure and function, inflammation, genetics, epigenetics, and sex-specific differences in frailty (48). One potential explanation for the higher prevalence of AD in women is their longer average life expectancy compared to men (49, 50). As of 2022, none of the AD clinical trials have specifically investigated sex differences in treatment efficacy or outcomes (51). However, recent studies have highlighted the critical neuroprotective roles of ovarian steroid hormones and their receptors in cognitive aging and AD development (52, 53). During the prodromal and mild cognitive impairment (MCI) stages of AD, circulating and cortical levels of ALLO decline sharply, which is associated with AD pathogenesis (54, 55).

## 4 Analysis of premenstrual dysphoric disorder as a potential risk factor for the development of Alzheimer’s disease

### 4.1 Role of estradiol in cognitive function, Alzheimer’s disease and premenstrual dysphoric disorder and its mechanisms

Ovarian hormones, particularly E2, are involved in many neurophysiological processes, including the maintenance of cognitive function.E2 plays a key role in the neurobiology of aging, as there is extensive interconnection between the nervous and endocrine systems (56). E2 is primarily produced in the ovaries, but it is also locally synthesized in other tissues, including the brain. Recent studies suggest that the brain itself is a steroidogenic organ (57). Estrogens are products of aromatase activation, a type of steroid hormone, which can act on estrogen receptors in both peripheral and brain tissues. The synthesis of estrogen begins in the mitochondria, where cholesterol is converted into pregnenolone. Through a series of steps, pregnenolone is converted into androstenedione, which is further converted into testosterone and estrone. Finally, through the action of aromatase (CYP19A), testosterone is converted into E2 (58). After menopause, as E2 levels decline, the incidence of cognitive impairment may significantly increase. However, women who maintain higher E2 levels perform better in executive functions compared to those who do not receive E2 therapy during menopause (59).

Some studies have found that E2-based therapy may help slow cognitive decline and could be a protective factor in the development of AD (60), Different hippocampal subpopulations are composed of various neuronal cell types, and their coordinated action generates distinct forms of network activity, which form the foundation for cognitive functions such as spatial navigation, learning, and memory (59, 61). In the hippocampus, the concentrations of sexual neurosteroids (SN) like 5α-dihydrotestosterone (5α-DHT) and E2 are significantly higher than those found in male and female serum (62). E2 enhances hippocampal object recognition and spatial memory, depending on the rapid activation of extracellular signal-regulated kinase (ERK) in the dorsal hippocampus (dHPC) (63). Estrogen receptors (mainly Estrogen Receptor α (ERα) and Estrogen Receptor β) are expressed in multiple brain regions associated with reproductive and cognitive functions (64). The ER-α gene (ESR1) is located on chromosome 6q25.1, while the ER-β gene (ESR2) is located on chromosome 14q22-24 (65). ERα and ERβ have different distribution densities in regions such as the hippocampus, amygdala, and hypothalamus. ERα is predominantly found in hypothalamic nuclei related to sexual behavior, while ERβ is more abundantly expressed in regions associated with cognition, such as the basal forebrain, prefrontal cortex, temporal and parietal regions, and posterior cingulate gyrus (66). ERβ has been shown to be involved in cognitive functions and is believed to promote learning and memory, neuroplasticity, and the regulation of neurotrophic factors (67)Large population studies have reported an independent relationship between ESR2 polymorphismsadolescent depression (68) and AD risk (69, 70). Fluctuations in hormone levels are considered a contributing factor to the development of PMDD. The impact of hormonal changes, particularly in estrogen and progesterone, may not act solely through the ligands themselves, but rather through their corresponding receptors (71). A study conducted by Huo et al. (2007) found significant differences in the genotypic and allelic distribution of four single nucleotide polymorphisms (SNPs) in intron 4 of the ESR1 gene between women with PMDD and control subjects (72). SNPs are among the most common forms of genetic variation in the human genome. Many occur in non-coding regions, where they can play a critical role in regulating gene expression, particularly by altering the binding affinity of transcription factors to their motifs or through changes in epigenetic patterns such as DNA methylation (71). Another study found a strong association between the PvuII-ESR1 gene polymorphism and depression in women. However, these findings remain preliminary due to the limited number of studies and inconsistent results, requiring further validation (73). PMDD is strongly associated with irritability, anxiety, depressed mood, anger, and somatic symptoms. Therefore, further studies are warranted to validate the association between the PvuII-ESR1 genotype and the PMDD phenotype (74). The role of E2 in the nervous system is highly complex and closely linked to cognitive function, emotional stability, and neuroprotection. In AD, prolonged decreases in E2 levels are thought to contribute to cognitive decline, while in PMDD, rapid fluctuations in E2 levels may underlie symptom manifestation. Furthermore, polymorphisms in E2 receptor genes may play a pivotal role in the susceptibility to both PMDD and AD.

### 4.2 The role of the GABAergic system in Alzheimer’s disease and premenstrual dysphoric disorder: mechanistic insights and therapeutic potential

GABA is the primary inhibitory neurotransmitter in the central nervous system (75), Research by Giovanna Carello-Collar et al. (76) demonstrated an overall reduction in components of the GABAergic system in the brains of AD patients, along with decreased levels of GABA in the cerebrospinal fluid (CSF), indicating that the GABAergic system is particularly vulnerable to AD-related pathology. GABAergic neurons exhibit a high degree of heterogeneity in terms of their morphology, electrophysiological properties, and molecular markers. Current research suggests that GABAergic neurons can be categorized based on the calcium-binding proteins and buffering molecules they express, with common subtypes including parvalbumin-positive (PV^+^) interneurons and somatostatin-positive (SST^+^) interneurons (77, 78). PV and SST neurons represent the major GABAergic neuronal subpopulations in the AD brain, accounting for 70% of the total, and are currently the most extensively studied subgroups.PV neurons primarily target the soma, proximal dendrites, and axon initial segments of pyramidal neurons, whereas SST neurons predominantly innervate dendrites (77). PV and SST neurons represent the major GABAergic neuronal subpopulations in the AD brain, accounting for 70% of the total, and are currently the most extensively studied subgroups.PV neurons primarily target the soma, proximal dendrites, and axon initial segments of pyramidal neurons, whereas SST neurons predominantly innervate dendrites (79). Aβ induces neurotoxicity in GABAergic neurons by interacting with the receptor tyrosine kinase ErbB4, which is encoded by the ERBB4 gene (Erb-B2 receptor tyrosine kinase 4). The ERBB4 gene is predominantly expressed in PV neurons, and its specific deletion in PV neurons significantly alleviates Aβ-induced memory deficits in hAPP-J20 mice (80).

Imbalance in the excitatory (E)/inhibitory (I) balance of neuronal network activity can lead to various neurological disorders. The emergence of excessive neuronal excitability in AD is considered one of the potential factors contributing to the rapid decline in cognitive abilities (81),Evidence currently suggests that E/I imbalance, representing the instability of glutamatergic (Glu - ergic) and GABAergic system synaptic inputs, is the underlying cause of brain dysfunction in AD (82, 83). AD patients and AD animal models exhibit excessive activity in hippocampal neurons, which is caused by dysfunction of GABAergic system interneurons (INs) (84–86). Experimental studies by Shu Shu (87)and colleagues indicate that PPswe/PS1dE9 mice (AD mice) exhibit depressive-like behavior and short-term spatial memory deficits as early as 9–11 weeks of age, Electrophysiological analysis reveals an E/I imbalance in the prefrontal cortex (PFC).This E/I imbalance is caused by a significant reduction in the number and activity of parvalbumin-positive interneurons (PV INs) in this region. Furthermore, optogenetic and chemogenetic activation of remaining PV INs effectively improved depressive-like behavior and rescued short-term spatial memory in AD mice. A proton magnetic (88) resonance spectroscopy study indicates that GABA levels in the cortical regions of PMDD patients significantly differ from normal controls during the menstrual cycle, suggesting the involvement of the GABAergic system in the pathogenesis of PMDD. During hormonal fluctuations, the adaptability of the GABA system is essential for maintaining the E/I balance. Inability to regulate the E/I balance is associated with changes in cognitive function, emotional shifts, and increased susceptibility to psychiatric disorders (89). The brain is highly responsive to changes in E2, progesterone, and ALLO, enhancing plasticity during hormonal fluctuations (90, 91), Due to the effects of ovarian hormones and their metabolites (such as ALLO), the activity of the GABAergic system changes during the hormonal transition period (92).The main symptoms of PMDD are emotional, not sensory, but neurofunctional abnormalities associated with this disorder even appear in the visual cortex. Changes in the neuronal E/I balance in the visual cortex of PMDD female patients are likely explained by increased excitability of principal cells and/or impaired synaptic excitability regulation (93). There is currently limited research on E/I imbalance in PMDD females, and the specific mechanisms need further investigation. Both PMDD and AD exhibit alterations in the GABAergic system and E/I balance disruption. In AD, dysfunction of GABAergic interneurons leads to cognitive impairment, while in PMDD, changes in the GABA system may be the underlying cause of emotional symptoms and cognitive fluctuations. Both conditions may lead to similar neurofunctional abnormalities due to excessive neuronal excitability and impaired synaptic regulation, Therefore, the importance of the GABA system in both disorders may provide insights for the development of therapeutic strategies.

### 4.3 The role and mechanistic insights of allopregnanolone in Alzheimer’s disease and premenstrual dysphoric disorder

**Figure 2:**
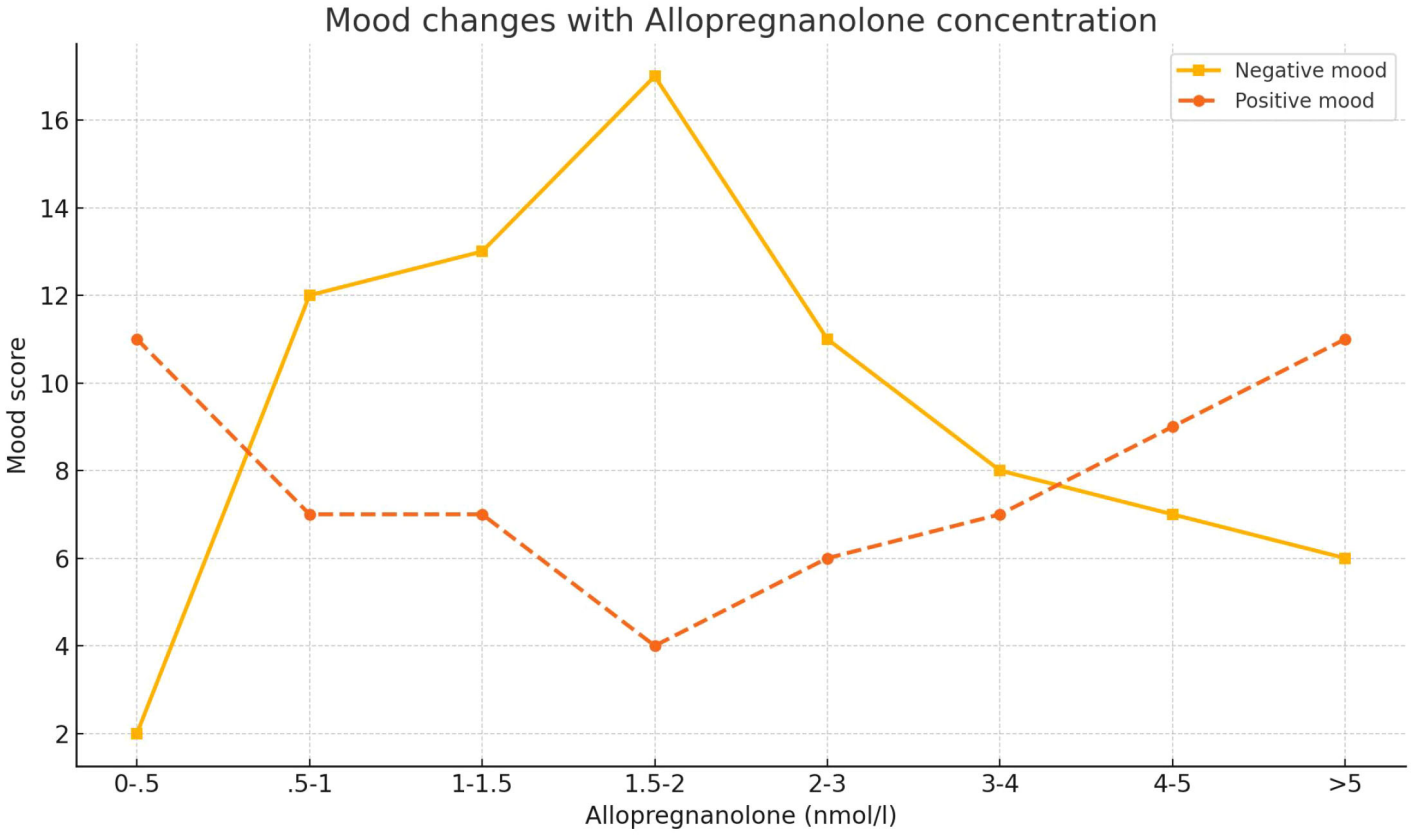
Dose-dependent effects of allopregnanolone on mood in postmenopausal women. The graph displays the mean ratings of negative mood (solid line) and positive mood (dashed line) across different serum concentrations of allopregnanolone (nmol/L). Data were derived from 37 postmenopausal women receiving estrogen and progestogen therapy. Mood ratings were collected on the same day as serum hormone assessments and grouped into eight allopregnanolone concentration intervals (n values per group shown in original figure). A U-shaped response is observed for both mood dimensions, indicating significant mood worsening at intermediate allopregnanolone levels. Error bars represent SEM. Statistical significance: *p < 0.05, **p < 0.01, ***p < 0.001. Adapted from Andreen et al. (2006b).

In the ALLO biosynthesis pathway, 5α-reductase first converts progesterone to 5α-dihydroprogesterone (5α-DHP), and then, 3α-hydroxysteroid dehydrogenase (3α-HSD) further converts 5α-DHP to ALLO. At the same time, 5β-reductase converts progesterone to 5β-DHP, after which 3α-HSD converts 5β-DHP to pregnanolone, which is another positive modulator of GABA_AR (94), The role of ALLO in the AD brain is closely related to brain cholesterol homeostasis. ALLO significantly reduces Aβ generation in the hippocampus, cortex, and amygdala, while also decreasing amyloid beta-binding alcohol dehydrogenase (ABAD) levels in mitochondria, thereby reducing microglial activation, as reflected by decreased expression of ionized calcium-binding adapter molecule 1 (Iba-1) (95), ABAD is a mitochondria-associated enzyme that promotes the reverse conversion of ALLO to 5α-DHP (96). Studies have found that ABAD is abnormally overexpressed in activated astrocytes, and its levels are higher in the AD brain (97). Furthermore, ALLO also regulates cholesterol homeostasis by influencing liver X receptors (LXR) and their associated pregnane X receptors (PXR) system (98), In APP/PS1 double transgenic mice, the deletion of either LXRa or LXRb subtypes exacerbates AD pathology (99), LXRs promote the expression of apolipoproteins by recruiting to the ABCA1 gene promoter region, thereby reducing the formation of Aβ plaques and enhancing Aβ clearance (95, 100). In addition, studies have shown that ALLO can induce increased expression of LXR in the pre-pathological state, while also increasing PXR expression in the brains of pre-pathological 3xTgAD mice (95), By activating PXR (mainly in neurons), ALLO further modulates the activity of cytochrome P450 3A (CYP3A) enzymes, including CYP3A4 and CYP3A13, ultimately promoting cholesterol hydroxylation and clearance (101). As AD pathology progresses, neurogenesis gradually decreases in AD animal models (including 3xTgAD mice) (101–104), particularly in the hippocampal SGZ of the dentate gyrus and the SVZ of the lateral ventricles, which is closely related to changes in cortical areas, including migration of the migratory flow in the septal region (105). Previous studies have shown that the production of ALLO can increase neuronal generation (106–108). ALLO significantly increases the number of newly generated cells and improves their survival rate, restoring the brain’s regenerative potential to normal levels (109). The regenerative effect of ALLO is dose-dependent, exhibiting a characteristic inverted U-shaped dose–response curve, indicating that higher doses do not necessarily enhance efficacy. At supraphysiological concentrations, ALLO induces sedative effects rather than promoting neurogenesis. From a safety perspective, the inverted U-shaped dose–response curve of ALLO is particularly critical. Supraphysiological or sustained high levels of ALLO may inhibit neurogenesis, thereby preventing uncontrolled cell proliferation (110). ALLO is currently under clinical development as a novel regenerative therapy for AD, with an upcoming phase II, multicenter, randomized, double-blind, placebo-controlled trial aimed at evaluating its efficacy and further assessing its safety (111).The emotional effects of ALLO in PMDD also exhibit a U-shape **Figure 2**, and blocking ALLO on the GABA_AR receptor represents a novel therapeutic strategy for PMDD. Sepranolone is a progesterone antagonist that can be administered via subcutaneous injection. In a randomized, placebo-controlled trial, bi-daily subcutaneous injections of sepranolone significantly improved the severity of PMDD symptoms, with its efficacy similar to that of SSRIs and combined oral contraceptives (COCP) containing drospirenone (34).

Given ALLO’s dual role in PMDD and AD pathophysiology, future research should focus on its clinical potential as a neurosteroid modulator. Specifically, longitudinal monitoring of cognitive function and neurobiomarkers in PMDD populations is recommended to evaluate whether ALLO analogs can prevent or delay AD onset. Moreover, integrating neuroimaging and biomarker analyses may help optimize ALLO dosing strategies to mitigate adverse effects from suboptimal concentrations. In addition, the combinatorial use of ALLO with other treatments (e.g., SSRIs, hormone therapy(HT)) warrants further investigation for potential synergistic effects.

In summary, ALLO serves as a critical molecular link between PMDD and AD, with considerable therapeutic and preventive value. Future clinical studies should focus on its dual-modulatory mechanism, facilitating innovative intervention strategies that span from mood disorders to neurodegenerative diseases, thereby offering more effective therapeutic options.

### 4.4 The role of serotonin receptor subtypes in premenstrual dysphoric disorder and Alzheimer’s disease mechanism overlap and research gaps

With the continuous improvement of diagnostic strategies for prodromal AD and the lack of effective early interventions, there is an urgent need to explore more proactive treatment strategies. Given the increased anxiety symptoms in the prodromal and preclinical stages of AD, targeting emotional regulatory circuits such as the hypothalamic-pituitary-adrenal (HPA) axis may offer a promising therapeutic direction (112); HPA activity is regulated by 5-HT (113). 5-HT primarily acts on G protein-coupled receptors and is a key signaling molecule in neuroactive ligand-receptor interactions, serving as an important central neurotransmitter (114). In AD patients, the expression and function of 5-HT receptors exhibit complex and region-specific changes, which further exacerbate cognitive and neuropsychiatric symptoms. Additionally, the dorsal raphe nucleus responsible for 5-HT synthesis in the AD brain shows marked neuronal loss and dense neurofibrillary tangles (115). 5-HT receptors are typically divided into 7 families and 14 subtypes (116), Among them, the 5-HT1A receptor is involved in the HPA axis stress response and is closely associated with anxiety, depression, cognitive changes, and psychiatric disorders such as schizophrenia (117). Activation of the serotonergic system has been shown to block Aβ oligomer-induced inflammation in AD, thereby affecting disease pathology (118). Compared with AD model rats, those treated with 5-HT1A receptor antagonists and 5-HT2A receptor agonists showed significant biochemical improvements, including reductions in brain inflammatory markers, oxidative stress, and Aβ deposition (119). Moreover, 5-HT6 receptor antagonists not only improve cognition in AD patients but also exert neuroprotective and anti-inflammatory effects by modulating neurotransmitter release and enhancing synaptic function (120). Xin-Rong Wu et al. (121) reported aberrant 5-HT receptor expression in the basolateral amygdala (BLA) prior to AD onset, with concurrent upregulation of 5-HT1AR in parvalbumin-positive interneurons (PV INs) and 5-HT2AR in pyramidal neurons, contributing to excitatory/inhibitory (E/I) imbalance and cognitive impairment. As a subregion of the amygdala, the BLA plays a central role in emotional and behavioral regulation (122). The amygdala (AMY) itself is a core node in emotional processing, and BLA hyperactivity has been associated with increased anxiety levels (123). Notably, BLA dysfunction has also been observed in PMDD patients (124), suggesting it may represent a shared pathological node between PMDD and AD. Serotonergic dysfunction is one of the proposed mechanisms underlying PMDD. A PET study found that in healthy women, 5-HT1A receptor levels in the brainstem increase during the late luteal phase, whereas no such increase was observed in PMDD patients (125). This suggests that PMDD patients may exhibit impaired dynamic regulation of the serotonergic system, particularly during the luteal phase, with diminished stress adaptation to hormonal fluctuations. This state-dependent dysregulation may underlie the recurrent mood symptoms of PMDD. Victoria Puig et al. (126) further showed that presynaptic 5-HT1A receptors in the raphe nuclei are excitatory, whereas postsynaptic cortical 5-HT1A receptors are inhibitory. Additionally, 5-HT3 receptor subtypes are implicated in depressive pathology (127, 128). The traditional Chinese medicine formula Shuyujiaonang has been shown to alleviate PMDD symptoms by reducing the expression of 5-HT3AR and 5-HT3BR (129).

Taken together, both AD and PMDD involve intricate regulatory networks of 5-HT receptor subtypes. In particular, 5-HT1A, 5-HT2A, 5-HT3A and 5-HT6A receptors are emerging as critical mediators of mood regulation, cognitive function, and neuroinflammation. The BLA, as a key region in emotion-cognition circuitry, may serve as a convergent site of pathology. Current evidence suggests spatial heterogeneity in receptor expression across brain regions and cell types (e.g., PV interneurons vs. pyramidal neurons), as well as dynamic interactions among receptor subtypes that collectively shape E/I balance. Therefore, integrating the functional dynamics of 5-HT receptor subtypes and their interactions over time holds potential for uncovering overlapping mechanisms of PMDD and AD, as well as for identifying novel therapeutic targets.

### 4.5 The pathological link between major depressive disorder and Alzheimer’s disease: neuroinflammation and stress starting from remenstrual dysphoric disorder

Recent studies suggest that PMDD may trigger Major Depressive Disorder(MDD) and subsequently increase the risk of developing AD, forming a potential pathological pathway of “PMDD → MDD → AD”. The mediating mechanisms underlying this association primarily involve neuroinflammation and chronic stress responses. Typical symptoms of MDD include depressed mood, loss of interest or pleasure, and, in severe cases, thoughts of self-harm or suicidal behavior (130). Studies have shown that individuals with MDD have a significantly increased risk of developing late-onset AD, with approximately one-quarter of AD patients also suffering from MDD (131). Neuroinflammation is a common feature in brain pathology and is recognized as a contributing factor in both AD and MDD (132, 133). Microglia, the principal innate immune cells in the brain, play a central role in initiating neuroinflammation (134), MDD patients exhibit chronically elevated circulating pro-inflammatory cytokines, which have been shown to be modifiable by anti-inflammatory treatments. Similarly, in the brain tissue of AD patients, activated microglia are densely localized around amyloid plaques, indicating a strong inflammatory response associated with Aβ pathology (131), Aβ peptides are core pathological agents in AD, formed by proteolytic cleavage of amyloid precursor protein (APP) (135, 136), Dysregulated production of Aβ1–40 and Aβ1-42, which can cross the blood-brain barrier, may contribute to structural brain abnormalities and cognitive decline (137). Under chronic stress conditions, Aβ production may become dysregulated, exacerbating neurotoxicity. Colaianna et al. (138) demonstrated that intracerebroventricular injection of soluble Aβ into 250–300g adult male Wistar rats led to increased immobility in the forced swim test, indicating depressive-like behaviors. This suggests that Aβ burden may not only drive AD pathology but also trigger affective symptoms.

Further studies have revealed shared neuroinflammatory features in MDD and AD, including aberrant Factor-alpha (TNF-α) signaling and impaired Brain-Derived Neurotrophic Factor (BDNF) and Transforming Growth Factor Beta 1 (TGF-β1) pathways. TGF-β1 is a key anti-inflammatory cytokine that exerts neuroprotective effects against Aβ-induced neurodegeneration and plays a vital role in memory formation and synaptic plasticity. Studies have shown that TGF-β1 levels are reduced in the plasma of MDD patients, correlating with symptom severity and treatment resistance (139). In a study by Sebastiano Alfio Torrisi et al., it was found that the SSRIs fluoxetine and vortioxetine exert therapeutic effects on non-transgenic AD animal models through a mechanism involving the upregulation of TGF-β1 levels. In mice injected with Aβ, a significant reduction in hippocampal TGF-β1 was observed, which correlated with memory deficits and depressive-like phenotypes, alongside marked decreases in the synaptic protein synapsin and postsynaptic density protein 95 (PSD-95). Fluoxetine and vortioxetine significantly increased hippocampal levels of TGF-β1, synapsin, and PSD-95 in Aβ-injected mice (140). This study provides experimental evidence for the neuroprotective role of TGF-β1 and reinforces the potential contributory role of MDD in the pathogenesis of AD.

According to DSM-5, both MDD and PMDD are classified as mood disorders (141), Clinically, PMDD is often considered a prodromal state of MDD, with significant overlap in affective symptoms and underlying mechanisms (142).Epidemiological research further supports this link: a study by HARTLAGE S A et al. (143) found that 7 out of every 8 women diagnosed with PMDD developed MDD within two years, suggesting that PMDD may serve as a potential precipitating factor for MDD. A recent study investigating the relationship between PMDD and inflammatory markers established a PMDD rat model using the Forced Swim Test (FST), and found elevated expression of TLR-4, nuclear factor kappa B (NF-κB) p65, TNF-α, IL-6, and IL-1β in the basolateral amygdala (BLA) of the model group compared to controls (124). These findings indicate abnormal activation of central inflammatory pathways under the pathological condition of PMDD. Ming Cheng et al. (144) systematically reviewed that stress-induced neuroinflammation constitutes a key pathogenic mechanism underlying the development and progression of PMS/PMDD. Given the critical role of inflammatory pathways in the pathophysiology of PMDD, further investigation into its association with other mood disorders and neurodegenerative diseases has become a major focus of current research.

Both MDD and AD involve chronic neuroinflammation as a central pathological feature and are currently focal points in neuropsychiatric research. Given that PMDD also exhibits elevated pro-inflammatory markers, it can be hypothesized that PMDD may act as an “inflammatory initiator” in the progression of mood disorders—first contributing to the onset of MDD, and subsequently exacerbating the neuroinflammatory milieu and disrupting neurotrophic factor signaling, thereby promoting the development of AD. Therefore, integrating the connections among PMDD, MDD, and AD from the perspective of stress and inflammatory pathways may not only help elucidate the progression mechanisms of female-specific neuropsychiatric disorders, but also provide novel targets for early screening and individualized intervention in AD.

### 4.6 Supplementary discussion of bidirectional causality: potential reverse impact of early Alzheimer’s disease pathology on premenstrual dysphoric disorder

Although this study focuses on the pathological pathway from PMDD to MDD to AD, it is noteworthy that core AD pathological changes, including Aβ deposition and pathological tau, may occur decades before clinical symptoms emerge (145). These early alterations may disrupt central nervous system function, thereby interfering with emotional regulation circuits and endocrine axis stability, potentially leading to cyclic mood disturbances resembling PMDD. Furthermore, existing literature indicates a bidirectional relationship between MDD and AD: on one hand, depression is considered a major risk factor for AD; on the other hand, early AD pathology may induce anxiety- and depression-like symptoms (146). PMDD, characterized by cyclic mood fluctuations, may be influenced by similar mechanisms. In other words, a subset of women with PMDD may exhibit abnormal hormonal responses and emotional reactivity due to early Aβ-related brain pathology. Future research should specifically examine whether early AD biomarkers (e.g., Aβ PET, cerebrospinal fluid p-Tau, abnormal TGF-β1) are present in women with PMDD, to determine whether PMDD represents a prodromal manifestation of AD in certain individuals. Exploring this direction may not only enhance our understanding of the disease spectrum of neuropsychiatric disorders but also provide new insights for sex-specific screening and intervention strategies for AD.

### 4.7 Sex-specific effects and neuropathological roles of APOE genotypes in Alzheimer’s disease and premenstrual dysphoric disorder

APOE is considered the strongest genetic risk factor for sporadic Alzheimer’s disease (AD), influencing both the average age of onset and the lifetime risk of developing the disease. The APOEϵ4 allele markedly increases the risk of AD (147). In the pathogenesis of AD, the sex-specific effects of APOE are particularly notable, with women generally exhibiting higher incidence and lifetime risk compared to men (148) Female APOEϵ4 carriers show the highest risk and tend to develop the disease at an earlier age (149, 150). Additionally, sex differences in AD risk may be related to early developmental processes of sexual differentiation, which predispose women to a higher susceptibility to AD and related dementias (151). At the biomarker level, studies have shown that female APOEϵ4 carriers exhibit higher levels of AD-related biomarkers in CSF (152) along with reduced hippocampal volume (153); Regarding Aβ pathology, APOE genotypes influence cerebral Aβ deposition in a dose-dependent manner (ϵ4 > ϵ3 > ϵ2) (154–156), and its regulation of tau-related pathology appears to be independent of Aβ (157). Christina B. Young et al. (158) demonstrated that APOE genotypes differentially affect regional tau burden during early AD pathology, with ϵ4 being specifically associated with elevated tau in the medial temporal lobe (MTL), while the protective effects of ϵ2 are observed in both the MTL and neocortex. Sex further modulates APOE’s effects on tau pathology (152),and one proposed mechanism is the lifelong difference in exposure to sex hormones, particularly E2 (159, 160), Low E2 levels have been strongly associated with increased AD risk, and E2-based HT is considered a potential preventive strategy. Several case-control and prospective studies have reported reduced dementia risk in women who are current or past users of HT (161–163). Animal studies have further shown that E2 can suppress abnormal tau phosphorylation, with females exhibiting greater sensitivity. Notably, estrogen receptor alpha (ERα) has been found to colocalize with neurofibrillary tangles in neurons (164). ERα levels are elevated in AD patients (165), and have been linked to APOEϵ4 in EFAD mouse models (166).

It is also noteworthy that APOEϵ4 is associated with an increased risk of depressive disorders (167), Considering that PMDD is an estrogen-fluctuation–sensitive mood disorder closely associated with changes in estradiol (E2) levels and exhibits a high risk of depression, there may be a potential interaction mechanism between APOE and PMDD. Specifically, female carriers of the APOEϵ4 allele may exhibit heightened hormonal sensitivity and increased reactivity to E2 fluctuations, thereby exacerbating emotional instability. Although current research lacks systematic data on the relationships among E2 levels, brain function, and mood disorders in women with PMDD carrying APOEϵ4, preliminary evidence suggests the existence of such mechanisms. For example, increased emotional susceptibility associated with estrogen receptor gene polymorphisms (e.g., ESR1) has been observed in the PMDD population, which may synergize with the APOE pathway.

Future studies should focus on the dynamic changes in sex hormone levels, polymorphisms of estrogen receptor genes, and their roles in emotional regulation in APOEϵ4 carriers with PMDD. Such efforts aim to identify high-risk female populations and explore potential biomarkers for early screening of neuropsychiatric disorders. Investigating this area will not only help elucidate the molecular underpinnings of PMDD but may also offer novel perspectives for identifying a subgroup of women at elevated risk for developing AD.

**Figure 3:**
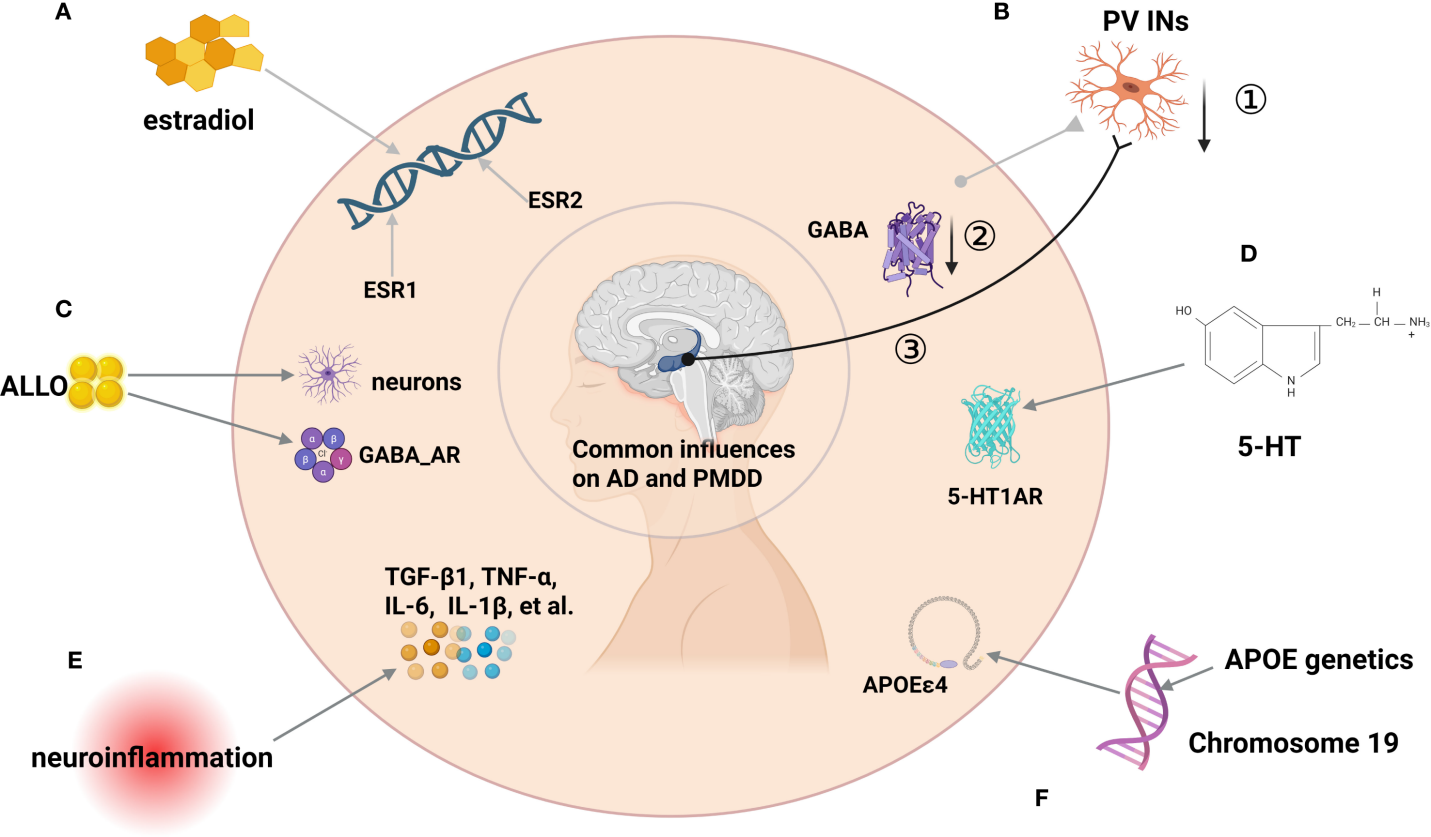
Schematic diagram of factors influencing the development of PMDD and AD. **(A)** ESR1 and ESR2 Genetic polymorphisms as factors influencing AD and PMDD.**(B)** ①PV INS affects E/I imbalance through reduced numbers,② reduced GABA synthesis and release, ③ and abnormalities in AD-related brain networks (e.g., impaired hippocampal-cortical loops) partially connected to GABAerqic INs. **(C)** Concentrations of ALLO have important effects in PMDD mood treatment and AD neurogenesis. **(D)** 5HT1R can affect PMDD and AD. **(E)** Neuroinflammation: In MDD and AD, anti-inflammatory TGF-β1 is reduced while TNF-α, IL-6, IL-1β rise with microglial activation (around Aβ), suggesting a pathway linking PMDD-related stress to AD. **(F)** APOEϵ4 genotypes can influence PMDD and AD.

Therefore, the intersection of APOE, E2, and female mood disorders underscores the necessity of incorporating PMDD into the early screening framework for neurodegenerative diseases and advancing prevention and intervention strategies based on sex and genetic risk profiles (**Figure 3**).

## 5 Conclusion

Although this study proposes a potential pathway linking PMDD and AD, it is important to emphasize that most of the current evidence remains correlational. There is still a lack of direct causal evidence supporting PMDD as an independent risk factor for AD. This limitation reflects one of the core challenges in current research. Longitudinal cohort studies, prospective clinical follow-ups, and mechanistic animal experiments are urgently needed to systematically track cognitive trajectories and dynamic changes in AD-related biomarkers in PMDD populations, in order to rigorously assess whether a causal relationship exists between the two. If this hypothesis is confirmed, it could provide a new framework for early detection and personalized intervention for AD, especially among high-risk female populations, with significant translational potential. Future studies should continue to explore the dual roles of neurobiological mechanisms in PMDD and AD, particularly regarding neuroactive steroids, GABA receptors, and the serotonergic system. A deeper understanding of these mechanisms may offer more compelling evidence to support the development of novel therapeutic strategies, alleviating symptoms and improving quality of life for affected individuals.
